# Ocular aberrations after wavefront optimized LASIK for myopia

**DOI:** 10.4103/0301-4738.64139

**Published:** 2010

**Authors:** Prema Padmanabhan, Subam S Basuthkar, Roy Joseph

**Affiliations:** Medical Research Foundation, Sankara Nethralaya, 18 College Road, Chennai, Tamil Nadu, India

**Keywords:** Induced aberrations, post LASIK higher order aberrations, wavefront aberrations, wavefront optimized LASIK

## Abstract

**Purpose:**

To study the change in ocular aberrations after wavefront optimized (WFO) laser *in situ* keratomileusis (LASIK) for correction of myopia and to analyze causative factors that may influence them.

**Materials and Methods:**

This was a prospective case series. WFO LASIK was performed for the correction of myopia, using the hansatome (Bausch and Lomb) microkeratome to create the flap and the Allegretto laser (Wavelight Technologie) to perform the ablation. The Allegretto wave analyser (Tscherning-type) measured the ocular aberrations prior to LASIK , one month and six months postoperatively.

**Results:**

The mean age of the 59 patients included in the study was 25±5.64 years and the mean spherical equivalent of the 117 eyes that underwent LASIK was ‒5.33±1.22 preoperatively and ‒0.21±0.38 postoperatively. Hundred and two eyes of 117 (87%) achieved uncorrected visual acuity (UCVA) of 20/20 or better after WFO LASIK and 104 of 117 eyes (89%) were within ±0.5D of the attempted refractive correction. There was a 1.96-fold increase in total root-mean-square of higher order aberrations. Induced changes in seven of the 22 higher order Zernike terms showed a significant linear correlation with the refractive correction attempted. Larger ablation zones induced less spherical aberration.

**Conclusion:**

In spite of an excellent visual outcome, WFO LASIK induces significant higher order aberrations. Large ablation zones reduce the induction of spherical aberration.

Excimer laser surgery is an effective method for the correction of spherocylindrical refractive errors of the eye. Although advances in technology were initially focused on improving the precision of refractive outcomes, recent efforts attempt to improve the optical quality of vision after such surgery.

The incidence of visual complaints by patients even after a successful refractive surgery, ranges from 3 to 40%.[1] These problems have been attributed to an increase in higher order aberrations (HOA)[2] following the procedure, due to multiple causes like flap-induced irregularities, decentration, variation in hydration of cornea during ablation and biomechanical changes resulting from laser surgery.

Studies have indicated that HOA of the cornea increase after radial keratotomy,[3] photo refractive keratectomy (PRK)[4] and laser *in situ* keratomileusis (LASIK).[5‐7] Although the surgical changes produced by refractive surgery occur on the anterior corneal surface, several groups have reported changes in the posterior corneal curvature after laser refractive surgery.[8‐10] It follows that the analysis of the total wavefront aberrations of the eye would provide the most direct and complete measurement of retinal image quality and therefore can be directly related to visual performance.[6]

Various studies have shown a significant increase in total ocular wavefront aberrations following refractive surgery.[11‐14] A comparison across studies is difficult because of the variability in the method of wavefront sensing adopted, the spot size and beam profile of the laser used, the ablation and transition zone diameters and the type and range of refractive errors treated. Most of these studies, however, reported the results of conventional corneal ablation, meant to treat only spherocylindrical errors, with ablation algorithms based on the paraxial formula of Munnerlyn *et al*.[15] These studies did show a significant increase in total HOA, particularly in the spherical-like and coma-like aberrations.[11‐14]

The Wavelight Allegretto excimer laser (Wavelight Technolgie, Inc., Erlangen) has a proprietary ablation algorithm that has a population-averaged spherical aberration correction built into it, and is referred to as “wavefront optimized (WFO) treatment". The WFO ablation profile was calculated based on subjective refraction and the amount of spherical aberration expected to be induced by conventional corneal laser surgery. The precompensation of this induced spherical aberration results in a larger amount of tissue removal in the periphery (approximately 35% more) than in the classic ablation profile, leaving the central ablation depth unchanged.[16] It does not take the unique aberration of each individual into account, and therefore cannot be termed a customized algorithm. However, it is meant to reduce the induced spherical aberration and thus improve the quality of the retinal image as compared to a conventional spherocylindrical correction.

This study aimed at characterizing the aberration profile of myopic eyes before and one month and six months after a WFO LASIK and studied the various surgical factors that could have influenced the changes induced by the procedure.

## Materials and Methods

This was a prospective study of patients who underwent LASIK for the correction of myopia or myopic astigmatism between January 2005 and June 2005. Eyes with any ocular pathology or previous surgery were excluded. Patients with any contraindication for LASIK (unstable refraction, keratoconus suspects, dry eyes, patients with collagen vascular disorders) were also excluded from the study. Patients willing to come back for follow-up visits one month and six months after surgery were enrolled in the study. All suitable patients signed an informed consent before being enrolled into the study which was approved by the institution review board and adhered to the tenets of the Declaration of Helsinki.

The ocular wavefront aberrations were measured by the Allegretto wave analyzer (WaveLight Technologie Inc., Erlangen AG) preoperatively, one month and six months postoperatively after pupils were pharmacologically dilated. Well-centered images with a grid pattern conforming to the criteria described in the Allegro Analyzer procedure manual were chosen for analysis. The Zernike coefficients up to the 6th radial-order were analyzed for a pupil diameter of 6 mm, and converted to the notation prescribed by the VSIA taskforce.[17] The appropriate algebraic conversion of all coefficients with an odd symmetry along the Y-axis in all left eyes allowed the data of both eyes to be analyzed together. The Hansatome (Bausch and Lomb) automated microkeratome was used to create a 9.5-mm corneal flap with a superior hinge (attempted thickness 160 µm).

The Wavelight Allegretto excimer laser (spot size 0.95) was used to perform the photoablation.

We defined “induced change” in aberration as the algebraic difference between the postoperative and preoperative Zernike coefficients, taking their direction (positive or negative) into account. We defined “factor of change” as the ratio of the “induced change” to the preoperative Zernike coefficient. The SPSS (Version 13) was used for statistical analysis.

## Results

LASIK was performed in 117 eyes of 59 patients (59 right eyes and 58 left eyes), with a mean age of 25 ± 5.64 years (range 18 to 35 years). Nineteen eyes had myopia and 98 had myopic astigmatism, with a mean refractive astigmatism of ‒0.85 ± 0.73 D (range ‒0.25 to ‒4.0D). The pre-operative mean spherical equivalent (SE) was ‒5.33 ± 1.22 (range ‒1 to ‒10D), and the best corrected visual acuity (BCVA) on the logMAR scale was 0.028 ± 0.139. The optic zone was 6 mm in 72 eyes, 6.5 mm in 41 eyes and 5.5 mm in four eyes. A residual stromal bed of at least 250 µ was retained at the end of ablation in all eyes. While all 59 patients came for a one month follow-up visit, 32 patients reported for a six months' evaluation as well.

At one month after LASIK, 102 of 117 eyes (87%) had an uncorrected visual acuity (UCVA) of 20/20 or better and 104 of 117 eyes (89%) were within +0.50D of the attempted refractive correction. The mean postoperative SE was -0.21±0.38 and the BCVA (logMAR) was ‒0.50+0.04.

**Figure 1:**
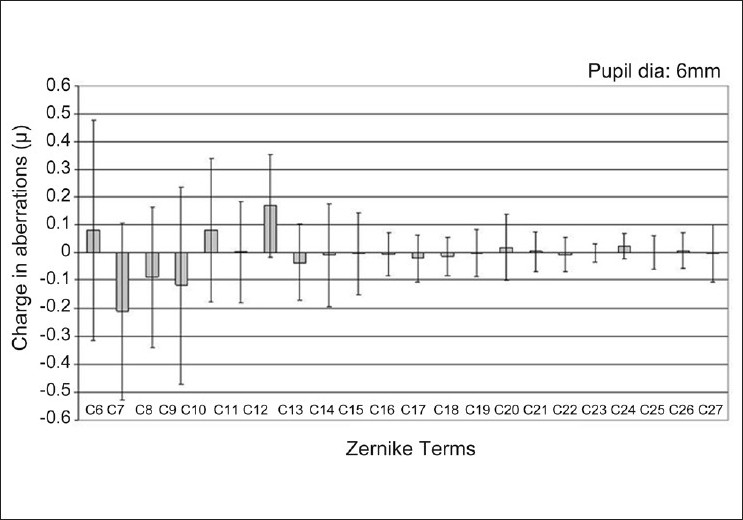
The induced change in Zernike coefficients from 3^rd^ through 6^th^ radial orders. Error bars denote ± 1 standard deviation

All the 27 aberrations showed a change from their preoperative values, and this was statistically significant (*P*<0.05) in 10 of 22 (48%) higher order Zernike terms (paired sample test). Fig. 1 shows the induced change in all the higher order Zernike terms from 3rd through 6th radial orders. Table 1 shows the mean induced change and factor of change seen in each of the Zernike terms of the 3^rd^ and 4^th^ radial orders. The largest magnitude of induced change was observed with vertical coma (-0.21±0.32) and with spherical aberration (0.17±0.19).

**Table 1:** Mean induced change (Postoperative from preoperative) and mean factor of change among Zernike terms of 3^rd^ and 4^th^ radial orders

Aberrations	Z^-3^_3_	Z^-1^_3_	Z^1^_3_	Z^3^_3_	Z^-4^_4_	Z^-2^_4_	Z^0^_4_	Z^2^_4_	Z^4^_4_
Mean Induced change (µm)	0.08±0.40	-0.21±0.32	-0.09±0.25	-0.12±0.35	0.08±0.25	-0.002±0.18	0.17±0.19	-0.04±0.14	-0.01±0.19
*P* value	0.03	<0.0001	0.0003	0.0005	0.001	0.89	<0.0001	0.007	0.57
Mean Factor of change	10.1	5.0	0.61	6.05	0.02	52.7	0.71	23.7	1.5
-*P* value	0.23	0.31	0.40	0.26	0.99	0.28	0.53	0.34	0.29

**Figure 2:**
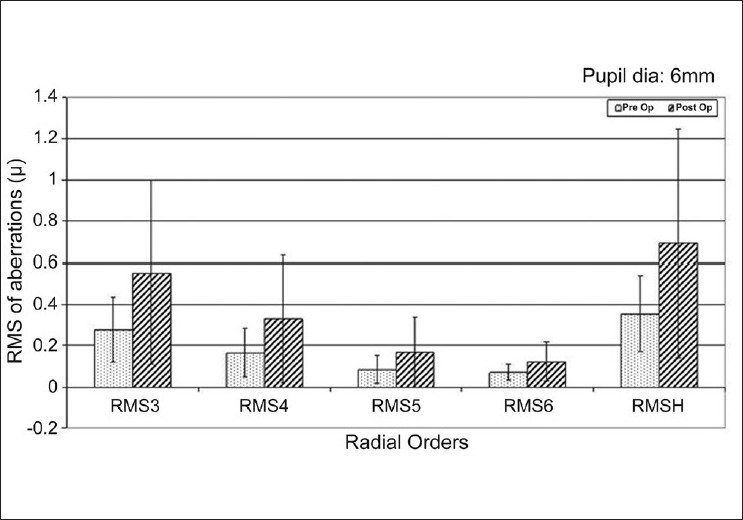
Root mean square (RMS) of 3^rd^ to 6^th^ radial order and total higher order aberrations before and after LASIK

Fig. 2 compares the root mean square (RMS) of HOA in each radial order from 3^rd^ through 6^th^ and the total HOA before and after LASIK. The RMS of total HOA following LASIK was 1.96 times that before LASIK. Table 2 shows the RMS of 3^rd^ and 4^th^ order aberrations and total spherical aberration (Z04 and Z06) and the ratio of postoperative/preoperative RMS.

**Table 2:** Root mean square (RMS) of 3^rd^ and 4^th^ order and total spherical aberration (Z^0^_4_ + Z^0^_6_) before and after LASIK and the ratio of postoperative to preoperative RMS in comparison with corresponding values from other reported results

Aberration	Pre-LASIK RMS (µ)	Post-LASIK RMS (µ)	Ratio post/pre Pupil = 6 mm	Barriuso *et al*. Pupil = 6.5 mm	Seiler *et al*. Pupil=7 mm
3^rd^ order	0.277±0.157	0.553±0.446	2.0	1.91	4.2
4^th^ order	0.165±0.117	0.328±0.308	1.99	2.10	4.7
Total spherical aberration	0.103±0.098	0.191±0.161	1.86	3.99	

*From Table 1 Barriuso *et al*. Invest Ophthalmol Vis Sci 2001;42:1396–1403.

The eyes were grouped according to the spherical equivalent of attempted correction into eight groups (<2D to >8D in 1D intervals). A linear regression analysis was done between the induced change in aberration of each individual Zernike term and each group of refractive error.

**Figure 3:**
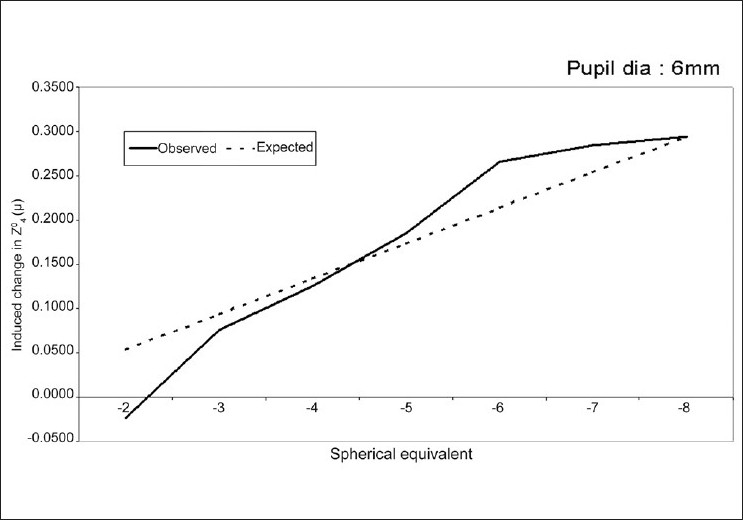
Linear regression graph between magnitude of induced spherical aberration and spherical equivalent of attempted correction

Seven of the 22 higher order Zernike terms showed a statistically significant linear relationship between the induced change in aberration and the attempted correction of refractive error [Table 3 and Fig. 3]. The induced change in RMS of total HOA also showed a trend towards an increase with increasing grades of myopia corrected [Table 4].

**Table 3:** Regression equations for the induced changes in the 7 Zernike terms that showed a statistically significant linear relationship with the spherical equivalent (SE) of attempted refractive correction

Zernike term	Regression equation	r value	*P* value
Z^-3^_3_	= -0.198-0.061×SE	-0.312	0.001
Z^-1^_3_	= -0.07+0.031×SE	0.197	0.037
Z^1^_3_	= 0.09+0.039×SE	0.312	0.001
Z^-4^_4_	= -0.155-0.051×SE	-0.405	<0.001
Z^0^_4_	= -0.026-0.040×SE	-0.483	<0.001
Z^-4^_6_	= 0.043+0.012×SE	0.358	<0.001
Z-^-2^_6_	= 0.014+0.003×SE	0.191	0.05

**Table 4:** The change in root mean square of higher order aberration from preoperative values (Δ RMSh) in relation to the spherical equivalent of attempted correction of refractive error, stratified in 1D intervals

Spherical equivalent	N	Mean Δ RMSh	S.D	95% C.I for Mean	
				Lower	Upper
0-2.00	8	0.18695	0.447064	-0.186805	0.560705
‒3.00 to ‒2.01	24	0.132673	0.21715	0.040979	0.224367
‒4.00 to ‒3.01	22	0.250717	0.482098	0.036966	0.464467
‒5.00 to ‒4.01	19	0.362194	0.352543	0.192274	0.532115
‒6.00 to ‒5.01	18	0.639696	0.808755	0.237511	1.04188
‒7.00 to ‒6.01	10	0.207964	0.318234	‒0.019686	0.435615
More than ‒7	12	0.648729	0.860841	0.101777	1.195681
Total	113	0.34032	0.551965	0.237438	0.443201

Seventy-two eyes had an optical zone of 6 mm and 41 eyes had an optical zone of 6.5 mm. Induced spherical aberration was statistically significantly less (independent t-test, *P* = <0.001) with the larger optical zone (0.08 ± 0.11 with 6.5 mm optical zone and 0.20 ± 0.19 µ with 6.0 mm optical zone).

There were 19 eyes with simple myopia which were treated with a spherical ablation profile and 98 eyes with myopic astigmatism who were treated with an elliptical ablation profile. A comparison of the induced changes in each group, however, showed no statistically significant difference.

**Figure 4:**
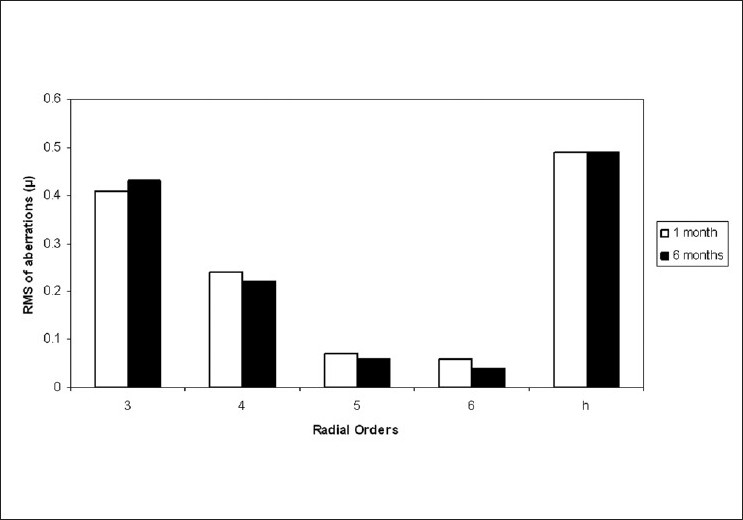
Root mean square of 3^rd^ to 6^th^ radial order and total higher order aberrations at one month and six months post-LASIK

Thirty-two patients returned for an evaluation six months postoperatively. Fig. 4 shows the mean RMS of their HOA at the one month and six months postoperative visits. There was no statistically significant change in the aberration profile in this period.

## Discussion

Several studies have shown a significant increase in corneal and total aberrations after laser refractive surgery. [1‐7] These, in turn, have been shown to correlate with a loss in low-contrast visual acuity and contrast sensitivity and with night vision problems like haloes, starbursts and glare.[2,7,18,19] An understanding and quantification of the aberrations induced by laser refractive surgery is an important prerequisite to formulate algorithms for customized laser procedures. While some studies have based their analysis on corneal topography data,[3,4,7] a few others have recognized that total ocular wavefront measurements are better predictors of retinal image quality.[11,12]

All the Zernike terms across the six radial orders measured showed changes in magnitude following LASIK, some more than others. Seiler *et al*.[11] reported a significant increase in four out of 22 higher order Zernike terms in 15 eyes after PRK. This study showed 10 out of 22 Zernike terms that had significant increase in the magnitude of aberrations after LASIK. The difference in procedure, limit of treated myopia and in the laser used, could explain this difference. Although some studies demonstrate that the creation of the LASIK flap induces significant HOA,[20] others report that most HOA after LASIK are induced by the ablation and not the flap.[21^,30^] Waheed *et al*.,[29] showed no statistically significant relationship between the position of the hinge of the flap and the induced coma and found that flap aberrations showed no predictable trends and accounted for less than a quarter of the increase in post-laser aberrations. Recent studies suggest that flaps created with the Intralase femtosecond laser were associated with less changes in HOA compared to the Hansatome flap.[22,23]

The ratio of postoperative to preoperative HOA [Table 2] was more comparable to the published results of Barriuso *et al*.[12] than those of Seiler *et al*.[11] Our results with the 4th order aberrations and with spherical aberration in particular, appear to be better than those reported by the others. Waheed *et al*.,[29] reported an increase in spherical aberration of 0.31 ± 0.08 at one to three months following conventional LASIK, whereas our study showed a 0.17 ± 0.19µ increase in spherical aberration one month following WFO LASIK. The ratio of postoperative to preoperative RMS of total spherical aberration (i.e. 4^th^ and 6th order spherical aberration) was 1.86 in our study (with pupil diameter 6 mm) compared to 3.99 in the study by Barriuso *et al*.[12] (with pupil dia 6.5 mm). Herein lies the unique feature of the WFO ablation profile, a proprietary algorithm of the wavelight Allegretto excimer laser, that pre-programs a population-based spherical aberration correction factor. Here, the ablation depth of the WFO ablation profile is up to 35% larger in the periphery than in the classic ablation profile.[16]

Our study, like some others,[6,12] showed a statistically significant linear relationship (*P* = <0.001) between the amount of induced spherical aberration and the refractive correction attempted. It was also influenced by the diameter of the ablation, being significantly less for larger treatment zones.

The increase in spherical aberration is a direct result of the change in corneal asphericity that occurs when the normal prolate shape of the cornea is changed to an oblate one. In addition, Dupps *et al*.[24] proposed that the biomechanical response of the cornea to laser photoablation for myopia could further induce spherical aberration. Marcos *et al*.,[6] showed, by using a combination of aberrometry and anterior corneal topography that a possible change in posterior corneal shape following LASIK would increase the negative internal spherical aberrations, and thereby attenuate the impact of the positive spherical aberration induced by the changes in the anterior corneal curvature.

Based on theoretical models, a few suggested ablation profiles could be considered to reduce the spherical aberration following LASIK.[25,26] These may be important in the context of wavefront-guided treatment. However, it is important to remember that biological, optical and mechanical effects will still induce unpredictable changes even if a perfect ablation is performed.

Our study showed a fivefold increase in the amount of vertical coma, and represented the highest magnitude of induced change among all the HOA. Unlike Barriuso *et al*.,[12] our study showed a linear correlation between induced coma and the attempted refractive correction although the correlation coefficient (r value) was only 0.19. There was no significant influence of the diameter or shape of the ablation zone on the magnitude of the induced coma. While Seiler *et al*.,[11] found that 4^th^ order aberrations dominated in postoperative eyes, our study showed that 3^rd^order aberrations continue to dominate after LASIK. There could be at least three sources of induced coma. First, even subclinical decentrations (less than 1mm) have been found to be a major factor in inducing coma.[1] Although the use of an eye tracker has helped to avoid severe decentrations, it does not ensure perfect centration.[27] Second, as suggested by Pallikaris *et al*.[20] the creation of a corneal flap with a microkeratome itself may induce coma along the direction of the hinge (superior hinge in this study), although the study by Waheed *et al*.[29] showed no predictable trends in flap-induced aberrations and no significant association between hinge position and coma. Porter *et al*.[21] demonstrated that while there was a significant sharp increase in vertical coma 20 min after the flap cut, this effect subsided in 24 h, suggesting that the postoperative coma reported universally in all studies, is due to the actual laser ablation and not the microkeratome incision, which was also the conclusion of the study by Waheed *et al*.[29] A third possible cause for postoperative coma could be an asymmetry in wound healing as suggested by immunohistological studies.[28]

Chalita *et al*.,[2] interestingly, found no correlation between vertical coma and optical symptoms, although horizontal coma was associated with double vision. This shows the importance of studying the orientation (positive or negative) of individual aberrations, rather than the RMS values alone.

LASIK induced a change in all the Zernike terms across six radial orders, affecting some Zernike terms more than others. The total HOA increased by a factor of 1.96. Vertical coma and spherical aberration showed the largest magnitude of change. The induced change in spherical aberration bore a linear relationship with the attempted refractive correction. Larger treatment zones were associated with smaller magnitudes of induced spherical aberration, but had no influence on the magnitude of induced coma.

Further studies on similar large datasets are required to confirm the occurrence and magnitude of such changes. The influence of biomechanical and wound healing on these changes also need to be further elucidated. This would help improve current treatment algorithms and result in better outcomes after LASIK.
